# Relationship between serum nitrite/nitrate levels in the early phase of septic acute lung injury and prognosis

**DOI:** 10.1186/cc11976

**Published:** 2013-03-19

**Authors:** M Sato, Y Suzuki, T Masuda, G Takahashi, M Kojika, Y Inoue, S Endo

**Affiliations:** 1 Iwate Medical University, Morioka, Japan

## Introduction

Serum nitrite/nitrate (NOx) levels in the early phase of septic acute lung injury (ALI)/acute respiratory distress syndrome (ARDS) have recently been reported to possibly play a key role in pathogenesis of ALI/ARDS.

## Methods

NOx levels in the early phase of septic ALI/ARDS were measured by autoanalyzer (TCI-NOX 1000; Tokyo Kasei Kogyo Co., Ltd, Tokyo, Japan). Cytokine was measured by ELISA (Medogenix, Fleurus, Belgium).

## Results

Both NOx and TNFα levels were significantly higher in the ARDS group than in the ALI group. A negative correlation was found between the PaO_2_/FIO_2 _(P/F) ratio and serum NOx levels. In addition, a positive correlation was found between the TNFα and serum NOx levels. The 30-day, 60-day and 90-day mortality rates were 8.7%, 15.2% and 19.6%, respectively, in the patients with ALI/ARDS. There were no differences in the P/F ratio, serum NOx levels or TNFα levels in the early phase of ALI/ARDS between the 30-day survival and death groups. On the other hand, the P/F ratio, serum NOx levels and TNFα levels in the early phase of ALI/ARDS were significantly higher in the 60-day and 90-day death groups than in the corresponding survival groups. There were no significant differences in the 90-day mortality rates between the ALI and ARDS groups.

## Conclusion

Our findings suggested that NOx may be involved in the pathogenesis of ALI/ARDS.

